# What are the experiences, preparation, and support needs of early career clinical educators within an Australian tertiary health service?: a qualitative study

**DOI:** 10.1186/s12909-024-05652-3

**Published:** 2024-06-16

**Authors:** Victoria Stirling, Deborah Fitzgerald, Alis Moores, Rachel Wenke

**Affiliations:** 1 Occupational Therapy, Gold Coast Hospital and Health Service, Queensland Health, Queensland, Australia; 2 Occupational Therapy Department, Townsville Hospital and Health Service, Queensland Health, Queensland, Australia; 3 Gold Coast Hospital and Health Service, Allied Health Research, Queensland Health, Queensland, Australia; 4 School of Health Science and Social Work, Griffith University, Queensland, Australia; 5 Faculty of Health Sciences and Medicine, Bond University, Queensland, Australia

**Keywords:** Allied health professionals, Clinical education, Early career, Educators, Novice, Occupational therapy, Placements, Students, Supervisor.

## Abstract

**Background:**

There is increasing demand for professional practice placement opportunities, supported by health professional educators, to enable future health workforce development. Early career health professionals performing the educator role is one strategy that can help meet this demand. However, there is a need to consider how best to prepare and support early career health professionals to become educators. This study aimed to explore the experiences and perspectives of early career occupational therapy clinical educators including their preparation and support needs.

**Methods:**

Semi-structured interviews were completed with ten early career occupational therapists who had supervised their first or second student on a professional practice placement. The participants worked within an Australian tertiary hospital and health service in various clinical settings. Interviews were completed within six weeks of placement completion and lasted approximately one hour. They were recorded and transcribed verbatim and reflexive inductive thematic analysis was undertaken to identify key themes.

**Results:**

Ten occupational therapists, who had been working for an average of two years and two months, consented to participate. Initially, participants expressed mixed emotions about taking on the clinical educator role. They then described their adjustment to the role responsibilities, challenges encountered, and the development of the educator-student relationship. Participants found that the experience of supervising a student enhanced their educator, clinical, and professional skills and confidence. The important support elements of tailored educator preparation, placement design, and timely access to relevant resources and experienced staff were identified.

**Conclusions:**

This study demonstrated how early career health professionals can possess desirable educator attributes, such as enthusiasm for taking on the role and cultivating collaborative learning relationships with their students. The experience of being an educator also presents a professional development opportunity for early career health professionals. Insights gained about the specific preparation and support needs of early career clinical educators warrant consideration by organisations and staff involved in the provision of student professional practice placements. Overall, this study’s findings signify the importance of engaging and investing in early career health professionals to support student clinical education and to develop our current and future healthcare workforce.

**Supplementary Information:**

The online version contains supplementary material available at 10.1186/s12909-024-05652-3.

## Background

There is growing pressure to source professional practice placements, herein referred to as placements, to support the development of future healthcare professionals. This stems from an increasing number of university health programs and student cohort sizes [1, 2]. Clinical educators, also referred to as fieldwork educators, practice educators, and clinical instructors [3], provide education and supervision of students during their placements. Therefore, it is essential to ensure that enough educators are available for placement provision, which can be a challenge. Sourcing placements and clinical educators is further impacted by the global healthcare workforce shortage, compounded by the impact of COVID-19 [4].

Early career health professionals performing the clinical educator role is one strategy that can help meet placement demand. However, the literature highlights that early career health professionals are still learning themselves and need to be provided with adequate support [5]. Being an educator involves additional responsibilities such as providing guidance, feedback, and assessment [6]. These responsibilities, combined with other well-documented challenges of being an educator, can lead to early career health professionals feeling apprehensive about taking on the role [7–9]. Ong and colleagues interviewed a total of twenty-nine occupational therapists and physiotherapists from two acute hospitals and a rehabilitation unit in Singapore and concluded that being an educator involved “overcoming feelings of unease and inadequacy” [10 p.1].

From the literature, various factors have been identified as worthy of consideration when engaging early career health professionals in clinical education. For example, Higgs and McAllister [11] raised the importance of educators adopting an active learning approach and reflecting on their role. In recognition of the time it takes to prepare for the educator role and support student learning, it has been suggested that educators would benefit from a reduced clinical caseload [9, 12]. The completion of formal and informal professional development activities before becoming an educator has also been recommended [13, 14]. This diverse range of factors indicates that the preparation and support of early career educators is not a simple systematic process and requires further exploration.

A health profession that has experienced significant global growth that is projected to continue and would particularly benefit from further inquiry is Occupational Therapy [15]. This growth of the profession has been particularly apparent in Australia [16]. However, to date, only one study has explored the experiences of early career occupational therapy clinical educators in the Australian context. Hunt and Kennedy-Jones’s [9] Australian interview study discussed how support from peers and more experienced staff, the ability to observe other staff with their students, and feeling comfortable within their clinical caseload were helpful for early career health professionals undertaking the educator role. Hunt and Kennedy-Jones [9] also identified that early career educators need support with the skill of assessing students. Despite offering some insights, this study was based on only four interviews and seven surveys of occupational therapists in rotational positions in a large metropolitan hospital.

There is a need for a more comprehensive qualitative exploration into the experiences of early career occupational therapy educators in different healthcare settings [9, 14]. This would better enable organisational supports, informed by an understanding of early career occupational therapy educator experiences, perspectives, and needs. The provision of organisational support to develop educator confidence and capability is essential for continued placement provision and the growth of the healthcare workforce. Therefore, this study aimed to investigate three research questions:

1. What are the experiences of early career occupational therapy educators in different practice settings in a large public tertiary health service?
2. What assists them in their preparation for taking on the educator role?
3. What are their support needs prior to and during a student placement?

## Method

A qualitative descriptive approach was chosen to explore the perspectives of early career occupational therapy educators to better understand their support needs. Ethical clearance was sought prior to the commencement of the study (HREC/2021/QGC/71,779) and the study is reported in accordance with the Consolidated Criteria for Reporting Qualitative Research (COREQ) guidelines for reporting qualitative research [17].

### Participants

Eligible occupational therapists worked in a single tertiary public health service and had up to four years of experience working in clinical practice. They also needed to have been an educator either once or twice for students on placements of five weeks or longer. Purposeful sampling was used to recruit participants working within a range of adult physical and mental health caseloads across hospital and community settings. Invitations to participate were sent via email within five weeks of placement completion. The email provided study information and consent details. A follow-up email was sent in cases of no response. The aim was to recruit ten participants with relevant experience. This sample size was considered sufficient due to the anticipated richness and value of the data obtained relating to the focused nature of the research questions [18].

### Data collection

The research team comprised of occupational therapists with research experience and discipline-specific knowledge (VS, DF, AM) and an experienced researcher of another allied health discipline (RW). The principal investigator (VS) was employed in a dedicated clinical education support position. For consistency, VS completed all participant interviews. VS was known professionally to the participants, which was considered beneficial for building rapport, but did not have line management responsibilities. Prior to the interviews commencing, a pilot interview was conducted, recorded, and reviewed by team members to refine the interview questions and technique. The semi-structured interviews utilised open-ended questions, supported by an interview guide (Appendix 1).

Interviews lasted approximately one hour and were conducted, where possible, face-to-face (seven participants) or via a Microsoft Team video call (three participants) within six weeks of the placement end date. All interviews took place in a private room at a workplace location convenient to the participant. The interviews were recorded using a mp3 audio recorder and transcribed verbatim. After conducting each interview, the principal investigator completed a reflective journal to enhance reflexivity. Participants were deidentified prior to data analysis. An audit trail of the analysis process was maintained to enhance credibility.

### Data analysis

Reflexive inductive thematic analysis of the interview data was undertaken following Braun and Clarke’s six-step guide [19]. This type of analysis was chosen due to the study’s aim to explore participant experiences, rather than apply a predefined framework. Key themes were developed and agreed upon by the research team aided by Nvivo software (version 12, QSR International Pty Ltd). VS reviewed the data, reading and re-reading the interview transcripts multiple times and electronically tagging and naming selections of text within each data item. VS then generated initial semantic data-driven codes and identified potential themes, based on repeated patterns in data content and interesting data content relating to the research questions. A second researcher (RW) reviewed all the initial codes and themes generated and assisted in further defining and refining the codes and themes through discussion and consensus with VS. These further refined codes and themes were then reviewed by the wider team to develop the final themes and subthemes. Consensus was reached through a process of iterative team discussion, supported by visual thematic maps and the research teams’ expertise in the research topic. All team members considered the overall validity of the final themes relating to the whole data set.

## Results

### Participant demographics and placement information

Eleven eligible participants were identified, of which ten were invited and agreed to participate. The remaining eligible participant left the health service shortly after placement completion and was therefore unavailable. The length of time participants had worked as clinicians ranged from one year and one month to three years and five months, with an average of two years and two months.

The majority of participants (*n* = 8) were first-time clinical educators, with the remainder (*n* = 2) being second-time clinical educators. The placements took place in various adult caseloads including acute, rehabilitation, and mental health services, in both hospital and community settings. Some of the participants (*n* = 4) shared the educator role with a more experienced clinician and all participants only educated one student per placement. The placements ranged in length from five to ten weeks, with the majority being full-time. Of the twelve student placements the participants supervised, ten students passed and two failed the placement.

### Educator’s perceptions

**Fig. 1:**
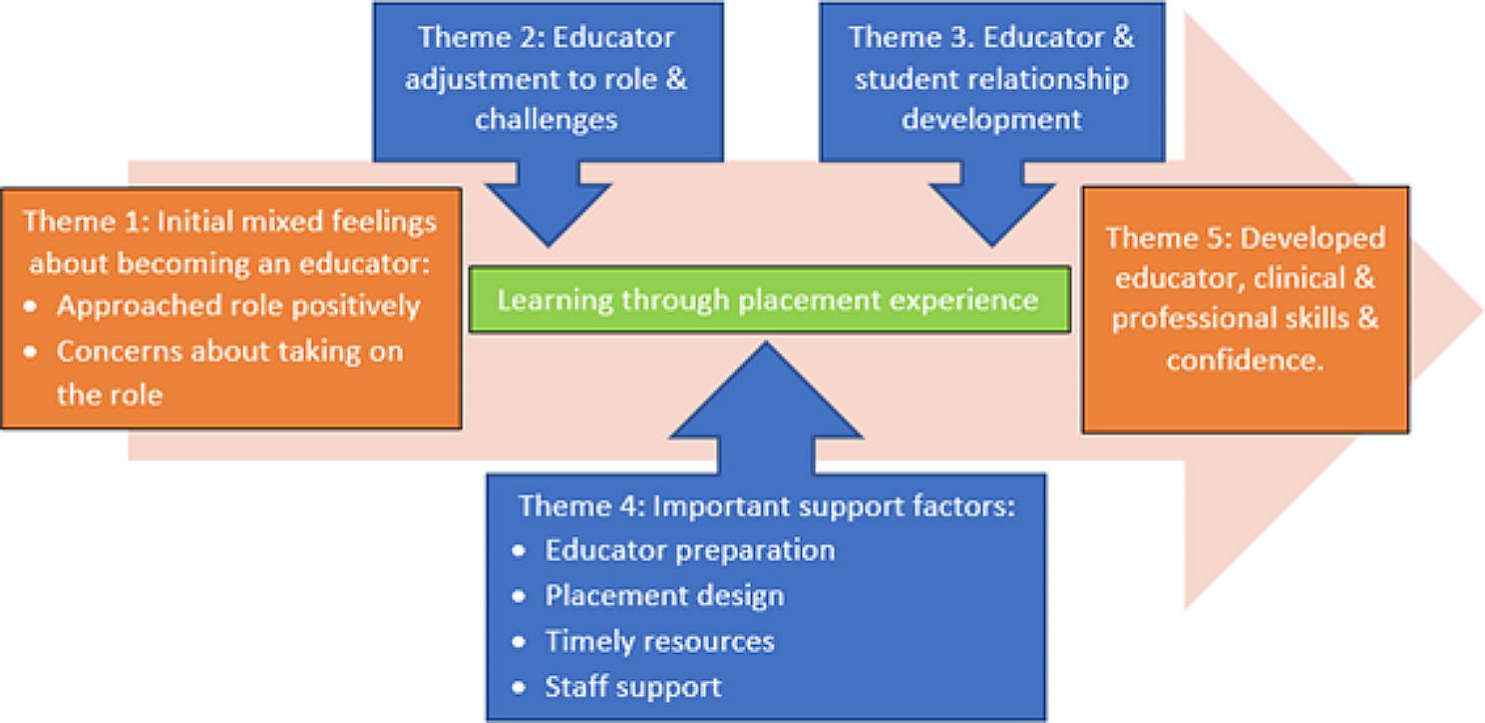
Early career clinical educator experiences and support needs

There were five main themes identified in the data as summarised in Fig. 1 together with subthemes. Initially, participants had mixed feelings about taking on the educator role (theme 1). They went on to describe how they learned through performing the role. This included adjusting to the responsibilities of the role, resolving challenges (theme 2), and developing the educator and student relationship (theme 3). Participants identified support factors they considered important (theme 4). Overall, through the experience of being an educator, participants reported improved educator, clinical, and professional skills and confidence (theme 5).

### Theme 1: Initial mixed feelings about becoming an educator

The mixed feelings expressed by participants were categorised into subthemes of how they of approached the role positively and their concerns about taking on the educator role.

#### Approached role positively

All the participants felt positive about taking on the educator role. Many of the participants stated they felt *“excited”* and viewed being an educator as *“a valuable learning opportunity” (P9).* Participants also appreciated the importance of providing placement learning opportunities to support students’ skill development. The notion that taking on the clinical educator role supported participants’ career progression was also expressed: *“I was excited to have a student to go the next step in my job as an OT [occupational therapist]” (P6).*

#### Concerns about taking on the educator role

Participants voiced concerns about balancing the educator role with their other clinical duties: *“I was nervous about how I was going to juggle my clinical tasks and the student” (P10).* The type of student they might be allocated also caused some “*hesitation or anxiety” (P7).* Many participants voiced feeling *“overwhelmed”* by the numerous placement resources available to assist them in the educator role. A common experience was participants feeling that they were still learning themselves. They described a lack of clinical and educator experience and confidence, with one participant admitting that she “wasn’t *confident really at all…. cause you just don’t know what to expect” (P1).* Another participant, referring to her student, questioned, *‘Do I know enough to give you a good placement experience? and am I going to be able to answer all your questions? …” (P2).*

### Theme 2: Educator adjustment to the role and challenges

Participants spoke of several aspects of the educator role that required them to adjust to a new way of working that they had not anticipated. One aspect was the need to frequently explain what they were doing and why: “*It’s just weird, having someone there all the time, making sure that I verbalise everything that I’m thinking about when we’re seeing a patient” (P5).* Additionally, the need to be continuously cognisant of where their student was and what activity their student was engaged in took some adjustment: *“I constantly felt that responsibility to make sure that they had stuff to do, or that I was telling them what I was doing so they were a bit aware and that whole aspect of it I found more draining than I thought, but I’d do it again easily, but I guess I just didn’t anticipate it” (P2 ).*

Many participants described how managing the *“competing demands” (P10)* of fulfilling clinical, non-clinical, and educator duties to be challenging at times. Simultaneously training and supporting new staff, caseload pressures due to staff absence, or students not performing as expected were added difficulties reported by some participants. The experience that it *“can be challenging just not having that space to yourself sometimes” (P9)* was shared by several participants. Overall, this need to adjust to the educator role and manage a variety of challenges was often described by participants to be tiring or hard.

### Theme 3: Educator and student relationship development

Despite the initial mixed feelings and challenges encountered, many participants reported that they enjoyed taking on the educator role, highlighting that the recency of their own student experience enabled them to relate to their students. They often described learning collaboratively with their students. One participant, when talking about learning work processes that she was unfamiliar with, explained *“you just kind of do it together” [referring to her student] (P2).* Participants also valued their student’s input with client care and appreciated *“having a sounding board and an extra set of eyes in difficult scenarios to help with gathering evidence…*” *(P6)*. Within the educator-student relationship, educators also often sought feedback from their students on their performance as educators: *“I’m more than happy for feedback for myself as well because that’s how we all learn” (P10)*.

### Theme 4: Important support factors

Four important support factors, or subthemes were identified from the data (Fig. 1). These included tailored educator preparation, placement design, access to timely resources, and staff support. These factors were interconnected, with one factor often reliant on the presence of another.

#### Educator preparation

When preparing to be an educator, the desire to feel *“comfortable”* and *“confident”* within their caseload before taking on the educator role was expressed by multiple participants. One participant explained this further: *“I was nervous about rotating to a new ward and having to familiarise myself while also familiarising my student as well” [to the caseload] (P8).* To prepare themselves for the educator role, many participants appreciated the opportunity to observe other health professionals with their students or take another health professional’s student for a limited time.

The preparation meeting with a dedicated clinical education support staff member was highly valued by all participants. One participant believed, echoing the response of others, that this meeting was *“a good opportunity to ask those questions that were burning in anticipation of having this [a] student*” *(P1).* Discussing the use and location of placement resources, placement details and processes, and student feedback approaches during these meetings was greatly valued. Participants also found discussion on how to approach the educator role with other more experienced clinical educators aided their preparation. Furthermore, attending a clinical educator workshop was considered most useful by participants shortly before placement commencement, to facilitate information recall and utilisation. Views on whether workshop content met learning needs varied among participants.

#### Placement design

Sharing the educator role with a clinician more experienced in clinical education was found to be helpful by all participants who experienced this approach: *“I think that’s a really nice way of stepping into that CE [clinical educator] role with sharing it with someone else” (P7).* The experienced clinicians guided the participants to set clear student expectations, provide feedback, complete the student evaluation document, and supported the participants’ workload management. Sharing the educator role also assisted the participants in reflecting on and developing their own educator skills. Although sharing the role was considered unanimously helpful, one participant spoke of the *“time-consuming” (P9)* nature of effectively communicating with each other when sharing the role. Another participant considered the impact of a student being exposed to two educator approaches to clinical care: *“we have different ways we do things” (P1).*

Participants found consistent caseloads and having patients with similar presentations that students could work with helped to facilitate student learning. Highlighting this point, one participant voiced *“I think because the clinical caseload is day in and day out quite similar you pick things up really quickly and you can run with it. And then you have that extra time to spend to educate and supervise and train someone else” (P1).* Incorporating quality student placement learning activities that were not dependent on the educator’s presence enabled the participants to balance work duties. Examples participants shared included student project work, self-directed learning, peer sessions with other students, or spending time with other staff: *“if there was a day … I needed to sit in the office and just get stuff done and get on top of things, not have to explain anything. We’d organise [student name] to go out …. with [colleagues name]” (P2).* They also appreciated the time and space away from their students that these activities provided: *“It was a nice balance for me to have breathing time on my own and doing my own thing” (P8).*

#### Timely resources

Various resources assisted the participants in performing the educator role and facilitating student learning, including resources that helped to communicate their expectations to their students: *“We used the student activity plan early on to set expectations about when we would expect [the student] to achieve certain things by” (P6).* Weekly supervision templates that linked to the overall student evaluation document were also appreciated and supported student goal setting, with one participant admitting that *“it’s really hard to keep track of everything, which obviously those supervision templates really helped” (P1)*. Additionally, “orientation checklists (P5)”, “tools to use for reflection” (P4), and a variety of treatment planning, feedback and project planning tools were also utilised. These resources came from a mixture of local, state-wide, or university sources. Frequently more experienced staff guided participants’ timely use of resources.

#### Staff support

Participants frequently reported needing support from staff more experienced in clinical education. This included dedicated clinical education staff, the clinician’s supervisor, peers, work team members, and university placement staff. This support assisted participants with educator skill development. Expectations for student learning, the provision of feedback, and the completion of the student evaluation document were common topics discussed. Participants appreciated receiving reassurance from experienced staff regarding their approach to facilitating student learning: *“[meeting with an experienced staff member] was really valuable, just to validate that I’m on the right track or just [get] some tips.” (P5).* They were also grateful for the emotional support the experienced staff provided. When referring to using their own supervision time during placement, one participant described it as *“just a place to debrief, sometimes it’s needed”* (*P9)*. Participants valued support from their work team to enable them to balance their workload. Helpful strategies of students spending time with other health professionals, team members, or team members assisting with the participant’s workload were identified by participants. This enabled *“protected time” (P8)* for participants to support student learning and complete the student evaluation document. Most participants reported feeling sufficiently prepared and supported in taking on the educator role.

### Theme 5: Development of educator, clinical, and professional skills and confidence

Over the placement, participants felt that their skills in tailoring their educational approach to their students’ needs improved. They spoke of their ability to adapt to their students’ learning styles and give their students the right level of responsibility to build their autonomy: *“…I think that initial just right challenge, I feel like I’ve started to navigate better” (P7).* There was a common theme of educators needing to learn how to “*observe and not intervene [during client interactions]” (P6)* to facilitate student skill development.

Furthermore, participants developed educator and professional skills in communication, time management, leadership, and provision of feedback. One participant, when asked whether there was anything that they felt they had improved in over the placement, responded by saying *“I think everything, just planning, giving concise feedback*, [and] *explaining things in a more concise way” (P4)*. Overall, participants felt that through the placement experience, their confidence as educators had improved, resulting in them feeling “a *lot more confident to be able to take on another student” (P9).*

Participants also described how teaching consolidated their clinical knowledge and skills: *‘’it reaffirms your own knowledge base” (P1).* It also prompted them to reflect on their learning and skills, as they wanted to do their best as an educator: “*when you’re having to teach someone, you go, wow, maybe I don’t know that as much as I should, and I should go brush up on that*” *(P9).* This self-reflection led them to seek supervision or support from more experienced staff: *“there were definitely a few things that I just wanted to confirm… so I felt confident in my competence” (P6)*. Some participants also described how they extended themselves clinically to provide more learning experiences for their students: *“my clinical skills definitely improved whilst [student name] was there because I’d sought out opportunities that before I wouldn’t have sought out.” (P2)*. Specifically, many participants identified that their clinical reasoning was enhanced through needing to explain and justify their intervention choices to their students: *“I just thought through, reasoned through things better” (P6).*

## Discussion

This study explored the experiences of early career educators in different practice settings in a large Australian public tertiary health service, including their educator preparation and support needs. The findings demonstrate that early career health professionals can make a valuable contribution to student learning on placement. It is also evident that the experience of being an early career health professional educator is beneficial for educator, clinical, and professional skill development. This study further highlights the wide range of preparatory and support activities that require consideration.

### Benefits of being an early career educator: contributing to student learning

One key finding from this study was that participants approached the educator role with overwhelming positivity. This attitude is conducive to an effective student-educator relationship and a welcoming learning environment [3, 20]. This positive approach contrasts studies describing clinicians’ reluctance to take students or the motivator being predominantly one of duty [21, 22]. Early career occupational therapists being motivated to educate students is encouraging for supporting the profession’s growth.

The early career educators in this study demonstrated a number of educator skills recognised in the literature to be important for effective student placement learning, including collaborative and reciprocal learning approaches [6, 20]. They also found they could relate to their student’s learning experience due to the recency of their own student experience. These educator abilities and approaches are comparable to the literature published on near-peer learning, when someone more junior receives education from someone more senior in the same field, which can have mutual benefits [23, 24].

Notably, participants proactively sought guidance on educator skills they perceived to be important. These skills included teaching skills, communicating expectations, and being a professional role model. These have been highlighted in the literature as desirable educator skills [3, 6]. Participants’ implementation of strategies to enhance these skills indicates their high level of insight into educator role requirements, as well as their educator capability. This appreciation of desirable educator skills and the drive to acquire them further confirms the valuable contributions less experienced educators can make to student learning.

### Benefits of being an early career educator: educator, clinical and professional skill development

Participants in this and similar studies described their experience in the clinical educator role as being a developmental process that benefits from interactive and reflective strategies [11] whereby educator skills and confidence grew through performing the role [9, 14]. The early career educators in this study felt their ability to build student autonomy and provide the appropriate level of challenge developed as the placements progressed. Developing these skills addresses the common difficulty of trying not to ‘jump in too soon’ and allows students greater autonomy, as voiced by participants in this and other studies involving early career health professionals [7, 14, 25]. Participants reported that their ability to set clear expectations, provide feedback, and adapt to student learning styles also improved. The development of these educator skills is of particular significance as they are recognised as traits of skilled educators [6, 20].

Additionally, being an educator enhanced the participants’ clinical reasoning abilities, an essential skill of any health professional. This particular benefit has not been well documented previously. By taking on the educator role, participants appeared to extend themselves clinically to offer greater student learning opportunities and utilised supervision to ensure their clinical competence.

Participants’ transferrable professional skills were also enhanced through the placement experience, including communication, time management, organisation, and leadership skills. Surprisingly, the development of these professional skills has received little mention in previous studies. Participants being prompted to self-reflect by taking on the educator role was evident in this and other studies [9, 25, 26]. Self-reflection is a core competency of health practitioners that is linked to the provision of high-quality, safe, and effective care [27]. The importance of early career health professionals developing this skill makes taking on the role of educator particularly valuable.

### Early career health professional educators’ needs: placement preparation, design, and support

Unsurprisingly, participants in this study needed to overcome educator challenges, many of which are well documented in the literature including time and workload management [13, 28, 29]. They also needed to adjust to educator role responsibilities, such as continuously being mindful of having a student to supervise, support, and educate. This involved frequently describing and explaining their thinking relating to client care to assist their student’s skill development. This need to adjust to the responsibilities of being a clinical educator has also been noted in previous studies [14, 30]. Consideration of placement preparation, design, and support factors helped participants fulfill their educator responsibilities.

In preparation for the educator role, the opportunity to observe other clinicians with their students was considered valuable, which reiterates findings from Hunt and Kennedy Jones’s study [9]. Designing a placement where the early career health professional educator shares the educator role with a more experienced clinician was found in this study, as in earlier studies [9, 31, 32], to be a useful introduction to taking on educator responsibilities. Placement caseload consistency and incorporating student learning activities not reliant on educator input were also identified as helpful placement design elements. Having timely access to staff experienced in education was greatly appreciated by participants. This staff support assisted with educator, clinical, and professional skill development, including completing the student evaluation document. Furthermore, the merit of having dedicated clinical education staff to facilitate educator preparation and development and provide timely relevant resources was unanimously acknowledged by participants in this study. This access to a variety of staff support may have contributed to participants in this study not reporting a fear of failing students, a concern that has previously been identified as an educator challenge [9, 32–34]. Overall, early career health professional educators need access to a support network in the lead-up to, and during placement provision.

### Limitations and future directions

Data collected in this study was specific to a small number of participants from one health profession based in one health service, where dedicated staff provided clinical education support. However, while some experiences may have been unique to the health profession and service, the majority of findings are consistent with current literature indicating potential transferability to other professions and services. Another potential limitation was the interviewer being known to the participants. While this was considered helpful for rapport building there is the possibility that this influenced participants’ responses.

More extensive exploration into the effectiveness of some of the educator preparation, placement design, and support strategies described in the present research could further guide their implementation. The placement design strategy of incorporating quality student learning activities that are not dependent on the educator’s presence has already been identified as warranting greater research and this study further supports this recommendation [21]. Additionally, future research could include interviewing early career educators at various stages of the placement to capture their responses in the moment, as opposed to recalling after the placement had finished. Exploring student perspectives of being supervised by early career health professionals could also be valuable for understanding the impact of the educator support provided. Broader insights on this research topic may also be gained by investigating the experiences of early career health professional educators outside of a tertiary health service.

### Practice implications

This study’s findings concerning early career health professional educators’ experiences and support needs suggest three potential practice implications for staff and services involved in student placement co-ordination and provision. Firstly, it is important to emphasise the benefits for the individual, service, student, and profession, when engaging early career health professionals in clinical education. These benefits include educator, clinical, and professional skill development, which support the delivery of safe, high-quality, person-centred care. Secondly, there is a need to focus on timely tailored educator preparation, provision of useful resources, and support from experienced staff. Thirdly, consideration should be given to the multiple factors of placement design that can aid educators’ confidence and role performance, such as sharing of the educator role, student self-directed learning activities, and caseload consistency.

## Conclusion

The study demonstrates that early career occupational therapists can make a unique and positive contribution to student learning on placement. It is evident that early career health professional educators have specific learning and support needs, including needing to adjust to the role responsibilities and overcome challenges. Access to timely support and well-designed placements enables early career health professional educators to successfully perform the role. Services are encouraged to invest in supporting early career health professionals as clinical educators and systematically reflect on how they approach educator preparation and placement planning to optimise current and future workforce development.

### Electronic supplementary material

Below is the link to the electronic supplementary material.

Supplementary Material 1

## Data Availability

Raw datasets are not publicly available to preserve individuals’ privacy. Further data information is available on request to the corresponding author (VS).
