# Supplementary material for: What are the experiences, preparation, and support needs of early career clinical educators within an Australian tertiary health service?: a qualitative study
Source: BMC Med Educ. 2024 Jun 16;24:664. doi: 10.1186/s12909-024-05652-3 (PMC11181623; doi:10.1186/s12909-024-05652-3)
Supplement: Supplementary file 1 — Supplementary Material 1 [file 12909_2024_5652_MOESM1_ESM.docx]

Interview Guide

1. Before taking your most recent student how did you feel about taking on the CE role?
2. How did you prepare for taking on the CE role?
3. Is there any additional preparation that you feel would have helped prepare you for your CE role?
4. Can you now tell me about your experiences as a CE during the placement?
5. Were then any (other additional) challenges you encountered?
6. What supports were available to you?
7. Are there any additional supports that you would have liked access to, either prior to or during the placement?
8. Did having a student impact your own growth and development?
9. If this was not your first student, then how did your experience of being a CE this time compare with your previous first CE experience? (only asked for CEs >1 student)
10. Are there any learnings as a CE that you would like to take forward with you to taking your next student?
